# Left Shoulder Pain After Routine Colonoscopy: An Unusual Presentation of Splenic Laceration

**DOI:** 10.7759/cureus.7755

**Published:** 2020-04-21

**Authors:** Karolina N Dziadkowiec, Peter M Stawinski, Dhruvil Radadiya, Aviv Katz

**Affiliations:** 1 Internal Medicine, University of Miami, John F. Kennedy Regional Campus, West Palm Beach, USA; 2 Internal Medicine, University of Miami, John F. Kennedy Regional Campus, Atlantis, USA; 3 Internal Medicine, East Tennessee State University, Johnson City, USA; 4 Internal Medicine, John F. Kennedy Medical Center, Atlantis, USA

**Keywords:** splenic haematoma, colonoscopy complications

## Abstract

Splenic injury is an uncommon complication following a colonoscopy procedure. Splenic laceration typically presents with post-procedural abdominal pain. We present a case of non-specific shoulder pain, following an uneventful routine colonoscopy and highlight the importance of maintaining a high degree of clinical suspicion for the general gastroenterologist.

## Introduction

Colonoscopy is considered a safe and widely utilized diagnostic and therapeutic procedure for the evaluation of large bowel diseases [1]. Approximately 11.5 million colonoscopies are performed in the United States on a yearly basis, and this amount is expected to increase as the population continues to age [2]. On average, 1.3% of patients present to the emergency department following outpatient colonoscopy procedures, with a chief complaint of abdominal pain, often caused by insufflation of gas during the procedure [3]. Rarely, post-procedural complications may include gastrointestinal bleeding, bowel perforation, and infrequently splenic injury [1]. Although an uncommon complication, splenic rupture due to colonoscopy remains a significant concern and is associated with high mortality rate [1]. We report a unique case of splenic rupture after routine uncomplicated colonoscopy.

## Case presentation

**Figure 1:**
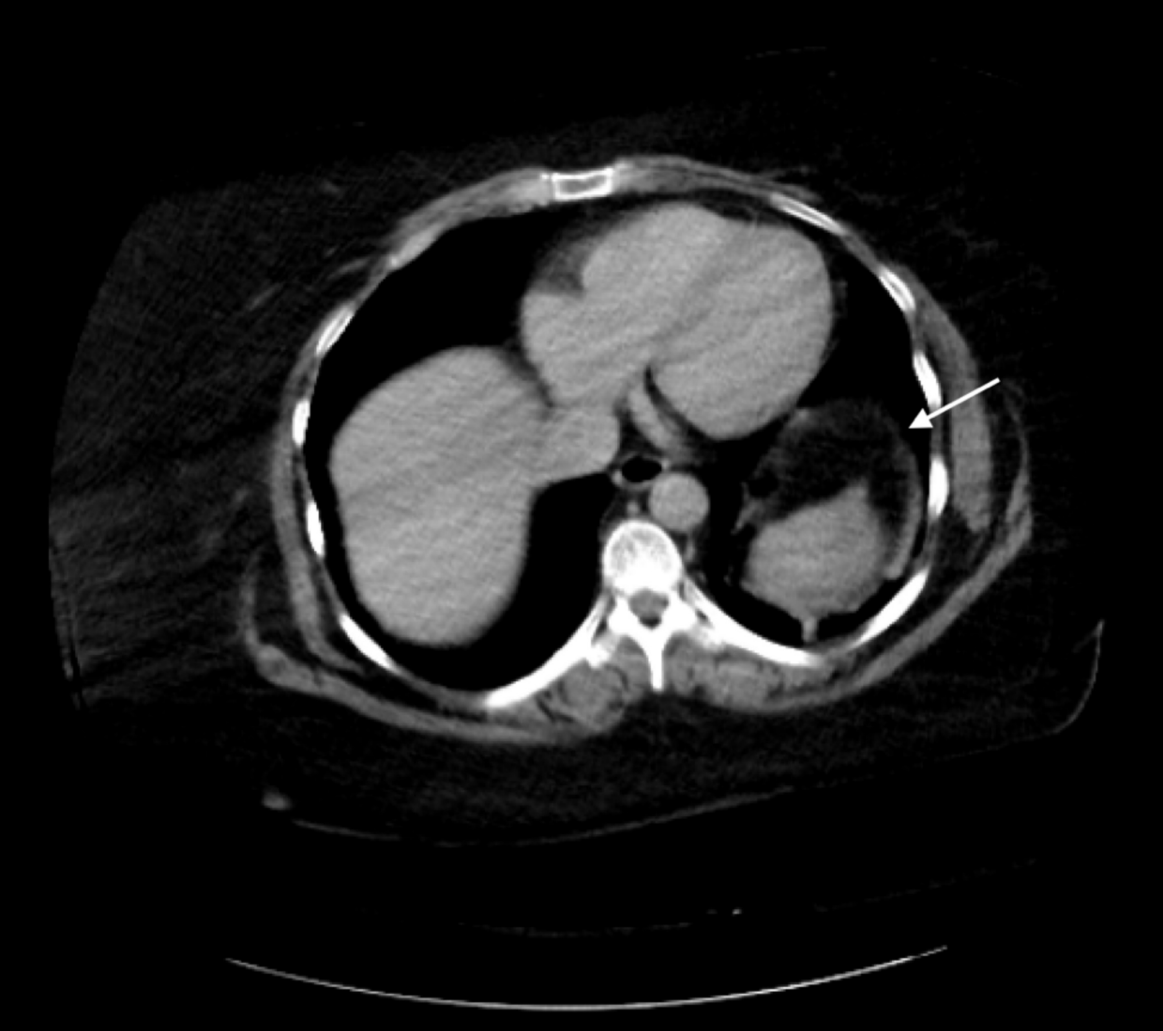
CT abdomen and pelvis consistent with large grade 3 splenic laceration and hematoma (white arrow)

A 49-year-old Caucasian woman, with asthma, gastroesophageal reflux disease (GERD), extensive smoking history and multiple abdominal surgeries in the past, presented to the emergency department complaining of severe left shoulder pain two hours following a scheduled outpatient screening colonoscopy. The colonoscopy was reported as uneventful and without complication. The patient was hemodynamically stable on admission. Hemoglobin was 13.5 g/L, hematocrit was 39.7 g/L and creatinine was at baseline; all other blood work was within normal limits. Vitals were stable and the patient was not in distress. Due to concern for post-procedural complications in the setting of recent colonoscopy, an urgent computed tomography (CT) scan of the abdomen and pelvis was performed which revealed an area of abnormal splenic hypoattenuation consistent with a hematoma and one band-like area extending approximately 5 cm into the parenchyma consistent with the American Association for the Surgery of Trauma (AAST) grade III splenic laceration without extravasation (Figure 1). The patient’s presentation was consistent with acute splenic rupture following colonoscopy. 

**Figure 2:**
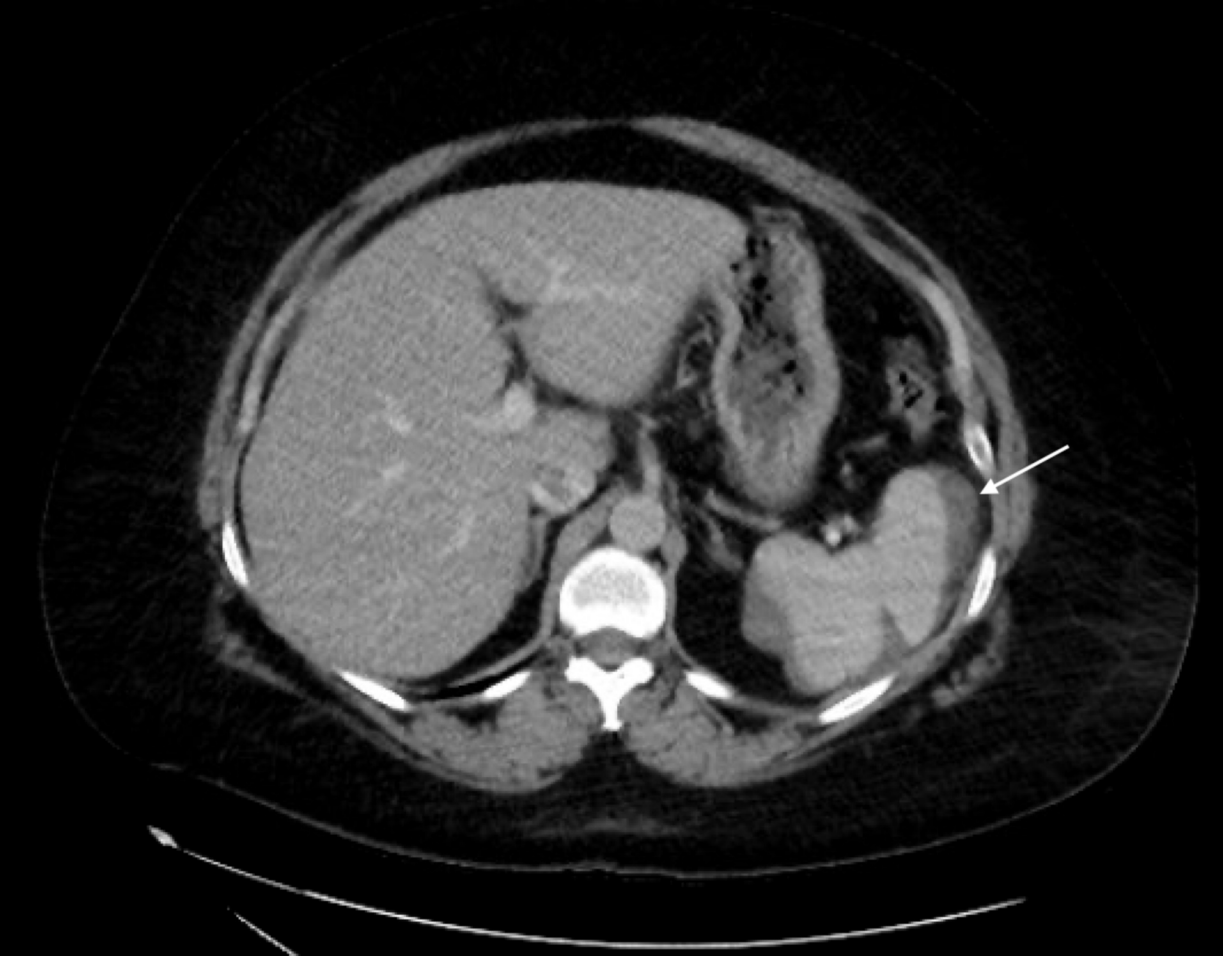
Repeat CT abdomen consistent with a pericapsular hematoma contouring the peripheral spleen appearing smaller and less dense

The patient was placed nil per os (NPO) and hemoglobin/hematocrit levels were followed closely and remained stable. The patient was managed conservatively with pain management and discharged after 48 hours of close monitoring and consistent hemodynamic stability. No complications were reported and the patient followed up three weeks after discharge. Repeat CT of the abdomen and pelvis at this time was consistent with a decrease in size of the hematoma and size of splenic laceration (Figure 2). The patient continues to be followed closely without further complications. 

## Discussion

In review of current literature, there are just over 100 reported cases of splenic injury following colonoscopy. Splenic laceration as a complication of colonoscopy was first reported by Wherry et al. in 1974 [4]. Its incidence has been reported to be approximately 0.00005%-0.017%, with a mortality rate of 5%. Splenic injury after colonoscopy is a relatively rare complication; a high index of suspicion is required when a patient presents following colonoscopy with abdominal pain or shoulder pain. The exact mechanism resulting in splenic injury is not entirely clear but likely related to direct trauma or excessive traction on the splenocolic ligament is believed to cause subcapsular microlacerations that may lead to overt laceration, rupture and hematoma formation [5-6].

The majority of patients become symptomatic 24 hours after their colonoscopy procedure. The most commonly reported symptoms include abdominal pain or discomfort located over the left upper quadrant, referred pain to the left shoulder also known as Kehr’s sign or non-specific symptoms of nausea, vomiting and abdominal distension [7-8]. Splenic injury after colonoscopy remains a relatively rare complication, it is often not the leading diagnosis considered after routine colonoscopy procedures and the diagnosis is often delayed. 

Our patient developed a grade III splenic laceration with large contained hematoma formation. A subcapsular hematoma is the most common injury pattern. The diagnosis is confirmed by clinical findings and complimented with CT imaging. CT abdominal scan is the preferred imaging modality, providing the most sensitive and specific qualitative data. Our patient’s unique presentation (with only left shoulder pain) reinforces the need for a high index of suspicion and awareness among general gastroenterologists for similar cases following standard endoscopic procedures. 

Treatment options vary, depending heavily on the hemodynamic status, splenic injury grading and associated injuries; patients can be managed conservatively with close observation, arterial embolization, or surgical intervention. AAST guidelines recommend urgent laparotomy for patients with hemodynamic instability and evidence of intraperitoneal hemorrhage or the presence of peritoneal signs on physical examination [9]. For hemodynamically stable patients, conservative management includes transfusions, splenic artery embolization therapy and monitoring of hemoglobin levels with serial abdominal examinations. A repeat CT scan is recommended to determine the extent of splenic injury and bleeding resolution [10]. 

## Conclusions

This case demonstrates that splenic laceration should be among the differential for patients who present with post-procedural complaints after colonoscopy. Such cases of splenic injury are often unreported or underreported and the complications of such occurrences go unappreciated. In addition, the colonoscopist should be aware that repositioning the patient, avoiding colonoscope looping, and minimal required sedation with careful monitoring of a patient's response during the procedure are important factors in reducing such complications. Splenic injury carries a high mortality risk, and prompt, accurate diagnosis can be lifesaving, therefore, cases such as this should be approached with a high degree of clinical suspicion. The general gastroenterologist should be aware of uncommon presentations of splenic laceration as a possible complication following routine endoscopic procedures. 
